# Medical Study; an Introductory Address Delivered at the British School, at the Opening of the Winter Session of 1836

**Published:** 1837-04-01

**Authors:** 


### MEDICAL EDUCATION.

Medical Study; an Introductory Address delivered at the Bristol Medical School, at the Opening of the Winter Session of 1836. By *J. A. Symonds*, M.D. Physician to the Bristol General Hospital, and Lecturer on the Theory and Practice of Medicine. Octavo, stitched, pp. 34. Bristol.

This Address is not unworthy of the fair professional fame of Dr. Symonds, nor of the medical school of Bristol. Delivered before the students of that school, it contains advice adapted to those both of province and metropolis, and to the students, also, of an older growth, who constitute the profession itself. At the present time, when young men engaged in learning medicine are seduced to political meetings, and withdrawn from the pursuit of what alone can ensure their success in after-life, to mix in discussions, and to nurture a spirit which brings shame on them now, and will entail future ruin—at such a time, it is pleasant to see a man like Dr. Symonds pointing out, with the force of reason and of eloquence, the true road to honour, fortune, and distinction. That road is broad, yet many miss it; and those who, disliking the "tolls" that are found on it—tolls which are indispensable for keeping it in order, ride "across country" on their sorry hacks, find to their cost that the near cut has carried them a great way round.

It must be obvious to all who watch the expression of public professional opinion, that a moderate party is daily acquiring more consistency and strength, and that its ranks contain the greater portion of the talent and the principle existing amongst us. That party is not limited to province or metropolis, to the graduates of this college or of that, nay, not to any grade in the profession. As the old aristocratical feelings and pretensions are gradually disappearing with the march of improvement, their possessors are fused with the mass of liberal, but moderate men, whose education enables them to see, and whose solid interests compel them to feel, that their places in society as gentlemen, and their rank in science, must be equally endangered by violent and disintegrating measures. It would be well, indeed, if our political arrangements could be once securely established in a mode satisfactory to all—it would be well if all could combine in advancing the interests of medicine by the peaceful and philosophic means which are congenial to it—but, alas, this seems an idle wish, an Utopian expectation—and the utmost that can be hoped for is, the consolidation of a party of moderate men—a party drawn from every rank—a party which shall oppose its calm sense and moral influence to the zeal of intemperate and the artifices of designing men. Dr. Symonds may fairly be said to represent the class of thinkers we allude to; and some of the passages we shall now extract embody the sentiments it entertains.

1. *Necessity of General Knowledge, and of a Scientific Character.*

Dr. Symonds observes, and with great truth, that the public expect from the medical man who aspires to distinction a considerable degree of learning, independent of what is strictly professional. It is because the physicians have possessed that in a higher degree than the surgeons, that they enjoy the superior relative rank—a noble ground for distinction. We have heard it said, that at the conversazioni of a celebrated whig nobleman, distinguished in politics and literature, a patron of science and the polite host of philosophers and littérateurs, physicians have always been cordially welcomed. The noble statesman has frequently observed that *their* society had ever proved amongst the most agreeable and most instructive, the least cramped by professional narrowness of mind, and the most tinctured by a liberal philosophy.

When we look back upon past ages, we have reason to be proud of the scholastic and literary merits of those who have left us immortal professional legacies. Hippocrates, Galen, Celsus, Aretæus, Linacre, Bacon, Sir Thomas Browne, Locke, Mead, Friend, Darwin, and Young, have reflected the scientific character of their age, or have even been in advance of it. That will be a dark day for the profession, when its general attainments are sunk in the mercenary struggle of interests and of necessities.

But it may be questioned whether it is possible for the mass of the profession to acquire the general knowledge that we speak of. It must be recollected that such knowledge is only obtained by a large sacrifice of time, and by a proportionate outlay of money—by an expenditure, in fact, of capital, which the majority have not, and which, *if* expended, would not bring, in the crowded ranks of general practice, an equivalent return. Medical assistance must be found for all classes. But, as all classes neither will nor can remunerate alike, those who attend the poorer cannot be expected, for the shabby income they receive, to disburse a great capital in the shape of general and preliminary education. This is the great regulator of professional ranks, this it is which we fear will disappoint, for the present, the amiable, and the desirable plan, of giving all practitioners high degrees, which can only be the reward of a monstrous outlay in previous medical and general study.

It has frequently been urged, and with all the intemperance of interest, if not of ignorance, that medical education is not cheap enough, and that greater facilities should be afforded to poor and industrious talent. A specious appeal to the best feelings of the heart, an appeal which is too apt to stifle the severe maxims of reason and of judgment.

But the man of the world is constrained to take society as it is actually constituted, and to acknowledge that many plausible plans of reformation are adapted for Utopia—but not for *it*. If all classes of men could be simultaneously improved, if abstract principles of justice and of virtue could be made to animate every breast, and to regulate every public and private action, if, in short, Great Britain could be transformed into Laputa, and her commercial sons into the sunbeam-sucking sages of that happy island, then many of our reformers might safely be permitted to legislate at large, and many of their propositions might reasonably be adopted. But that desirable event has not yet arrived, and, in 1837, the plan of cheapening medical education is exposed to one or two slight objections.

1. Different classes in this country are popularly considered to possess different degrees of what is called respectability. It so happens that the wealthier classes are usually deemed the most "respectable." Whilst these principles obtain (we do not defend them) a profession which offers no temptation for respectable people to join it, which can give them no prizes if they do join it, is scouted by them, and soon ceases to be thought respectable itself. Unfortunately, this is to a certain degree, the case with medicine at present. The bar, the church are both deemed more honourable in a worldly point of view than *it* is—not simply because the prizes they offer are higher, but because the capital necessary to enter either with a prospect of success is on the whole too great to permit the inferior classes to rush into them en masse. If we lower medicine, while the other learned professions are not lowered also in a proportionate degree, we necessarily increase the disparity between them, and degrade in the world's eye the former. Observe that we are simply stating the fact. We neither support nor condemn the public's mode of thinking. But the public do think so, and we leave it to the profession to decide whether it wishes to debase itself.

2. If medical men are agreed in any thing, it is in the conviction that they are too numerous already. This sentiment is in every-body's mouth. Ask any physician, surgeon, or general practitioner what he thinks the great practical evil, and ten to one he replies;—"The profession is overstocked." In all the large towns, nay in the very villages and hamlets, medical men, like the Kilkenny cats, are eating one another. If a doctor dies, two or three set up before the hearse has left the old man's door. We may exclaim with Hamlet—

> The funeral-baked meats
> Do coldly furnish forth the marriage table.

What a pregnant commentary on our crowded and uneasy state, has been lately read by the proceedings of the Commissioners of Poor Laws. They offered hard, almost insupportable terms to the old and established practitioners. Stung at the insult, smarting with the injury, the latter were compelled to close with the Israelitish bargain, because they knew, and, where they kicked at it, they felt, that nothing was so easy as for those Commissioners to procure, at a minute's notice, any requisite number of hungry, and not ignorant, scramblers for the bone they offered.

Is not this an evil? Poll the profession from the Lizard's Point to John o'Groats, and their wry faces will cry yes. And what is the remedy that our modern medical economists propose? Listen practitioners we pray you—listen to the marvellous panacea for your ills. They propose that every facility should be offered to the entrance of fresh members—that *poor talent* should be encouraged—that all "tolls" should be abolished, or, if not abolished, reduced to the very minimum. Admirable method of bettering our state! We are too rich—the whole profession is a monopoly—we require dilution—Homœopathic dilution.

Seriously, is it not quite evident that by rendering access to the profession very cheap, we offer a premium on the entrance to it? If we diminish the capital * necessary for a medical man much below that necessary for a lawyer or a clergyman, is it not clear that we shall have proportionally more medical men than clergymen or lawyers? The result is a ruinous competition—a competition already, with existing checks, too great—with diminished checks inevitably greater.

The cool calculator of the misery of the profession may apply to its case, the political principle of supply and demand. But that application will not bear the test of rigid and impartial scrutiny.

It is true that in commerce, capital will be embarked in proportion to the returns anticipated and obtained, and, if these returns prove inadequate, the capital, be it labour or money, or both, will sooner or later be withdrawn.

---

* We do not use the term capital in a monetary sense only—but as the representative of the time and money so expended in education.

But the great distinction between ordinary and professional capital is this—that the former is generally marketable and convertible, and the latter is not. If a merchant invests to-day 10,000 pounds in tobacco, and the profit is insufficient, or if a part of his capital is gone, he may put what remains in the funds, or he may embark it in the purchase of cotton, or in a thousand other manners. But if a man has been educated for our profession, and after some years' trial, fails of success, his professional knowledge is of no use to him in any thing else that he engages in, and that capital is entirely lost.

There is another evil, not at first so obvious, which must spring from the extreme cheapness of medical education. If we invite into our ranks large numbers of persons born and brought up in comparative indigence, their scale of artificial wants is so low, that they will be content to live on very little, or, rather, a very mean income indeed will not diminish but perhaps increase the comforts to which they have been previously accustomed. The necessary result is a grinding competition, the consequent diminution of the profits of all, and the degradation of the whole profession. Are medical practitioners so overpaid now, that they are anxious to encourage rivalry, increase their numbers, and generously lower their charges and returns? If they are, we must applaud the philanthropic spirit, and we recommend them to urge with augmented vehemence the abolition of all checks upon accessions to their ranks. True, they will grow poorer yearly—true, they will be snubbed by every commissioner, and every discontented patient—true, they will sink lower in the estimation of the world. But what of that? They will enjoy the proud satisfaction of knowing that if the fire burns less brightly in the hearth, philanthropy blazes in their bosoms—that if the purse becomes lighter, money is after all but trash—that if bullied by every jack-in-office, they have within them a noble independence, and a proper self-esteem—

> Which nought external can expose,
> To gross material bangs and blows.
> Then how is't possible a kick
> Can e'er reach that way to the quick?

that if the world thinks meanly of them, it erects a false and grovelling standard of respectability—in short, they will be told by liberals of the first water how patriotic and disinterested they are, and they will find what a charming thing it is to be so.

3. When we are presented with the picture of needy talent, knocking at the door of science, and spurned by the surly porter of wealthy monopoly, our indignation is naturally roused, and the generous impulse of our common nature is to throw those portals open to all. But reflection soon suggests a comparison between the evil and the consequences of the remedy, and forces on the attention that iron rule, which seems to regulate alike the maxims of Providence and man, *that* partial evil must be tolerated for the sake of the general good. The enthusiast looks only at one needy man of talent apparently unable to obtain what he merits. The philosopher regards the condition of a profession, the social welfare of many thousands. It would be well, if the utmost encouragement of talent were compatible with the general advantage of the mass—it would be well, too, if no such thing as moral or physical evil existed—if there were not pain, disease, decay—if there was no inequality amongst men—if the world was a Platonic commonwealth. But it is not yet so, and we must patiently await the moment when the efforts of the burning philanthropists about us will effect the happy consummation. Needy talent is at present in the situation of the knife-grinder, and requires to be stimulated by the "friends of humanity" to a sense of its unutterable wrongs.

> Needy knife-grinder! whither are you going?
> Rough is the road, your wheel is out of order—
> Bleak blows the blast; your hat has got a hole in't,
> So have your breeches!

No doubt there are some instances of talent oppressed by menial occupations, and unable to rise through the muddy waters that encompass it. Yet it may be doubted if in a country like this, *great* talent is frequently kept down. Difficulties are perhaps less unfavourable to its exercise and development than we might be led à priori to suppose; and it is certain that such difficulties have formed in many well-known instances, a stimulus to genius.

But, after all, the question reduces itself to this. Is it better to inflict certain injury on a profession, with the chance of picking up a Hunter or two additional—or, legislating for the mass, and in accordance with the general tone of society, to maintain the respectability and solid interests of that profession, by requiring from its introants what the very needy classes are unable to afford? This is the common sense view of the dilemma. It is for the profession itself to decide on it.

We will suppose that the profession is not willing to perpetrate felo de se, and that it *has* decided on protecting its dearest interests, and maintaining its respectability. We will suppose that it has refused to let in principles and measures which would inevitably swamp it, and send it water-logged down the stream of public opinion. We will suppose, in short, that the profession does not approve of the adoption of that "cheap and nasty" system, which would invite into its ranks all who could not hope to put foot within the pale of divinity and law, and would convert itself into a huge refuge for the destitute.

The question then arises—how give effect to this salutary determination? Two modes present themselves—either arbitrary tolls may be levied on the student, and monopolies erected for the taxation of him and the enrichment of themselves; or, an extended system of education may be enforced, and severe examinations may be instituted as a guarantee for its adoption.

The first proposal requires no consideration. Such tolls and such monopolies are contrary alike to the spirit of the times, and to the best interests of society, and neither would their erection nor continuance be tolerated. Legislation, whether supreme or municipal, must march with opinion, or will be trampled under foot. The only principle which it can recognize, or on which it can act, is the utilitarian one—"the greatest good to the greatest number." The sun of monopolies has set for ever.

But the plan of excluding crowds of paupers from our ranks, by requiring such a full measure of professional education as paupers cannot purchase, wears a very different aspect, and would be followed by very different results. It cuts two ways, and either way for good. It is a check upon the numbers who enter the profession, and it enhances the attainments, and consequently the respectability, of those whom it admits. The College of Surgeons and the Company of Apothecaries, have widely extended their curricula, and greatly elevated the standard of professional acquirements in the candidates for their diplomas. The competition of the various schools, their number, and their interests, preclude the idea of exorbitant fees, and, indeed, but for the lengthened period of medical education, that competition would be productive of the ruinous consequences we have dwelt on, the introduction of excessive numbers into our ranks.

There may, for aught we know, be some who will clamour at the principle of insisting on lengthened study and severe examinations. They will exclaim that this is monopoly too. Monopoly to a certain extent there is and must be. Those who cannot afford to comply with the curriculum are excluded. That is true enough, but frame what curriculum we may, adopt what restrictions we please, there still must be a barrier somewhere. Our most radical reformers never dreamt of abolishing a curriculum altogether. The question is only one of plus or minus, and its determination must rest, as we have already stated, on the principle of general utility.

We must now return to Dr. Symonds, from whom we have for some time parted company. He proceeds to inform his audience how knowledge is to be acquired, and he naturally enumerates three methods—reading, attending lectures, and personal observation.

2. *Mode of Professional Study.*

1. It is difficult to say to what extent reading should be carried, because it is difficult to lay down a rule which shall fit minds of various calibre. Dr. Symonds' observations on this head appear to us to be very just.

"Now I believe that on this, as upon so many other similar subjects, no rule can be devised, applicable to every description of intellect. There are some, for instance, who will derive from reading, and retain, what would have escaped them entirely had they been dependent solely on their powers of observation; while others have so little capacity for book study, and get so little by it, that time is lost upon it which might have been devoted to experience. But such extremes we must leave to shift for themselves, according to their respective peculiarities; I address myself to those who are able and willing to make use of either mean, according as it may promise more advantage. Reading, then, is never to take the place of experience; its object is to present you with that which, either you could not have learnt at all, or which, at all events, you could not have learned so soon, and with so little cost, without it. It is intended to give you the result of the observation of others, without the fatigue which they incurred in obtaining and in digesting it, and in making the proper inferences from it. Reading saves you a prodigious expenditure of time, even supposing you could have ascertained and discovered as much as the authors, under similar circumstances. It prevents you from repeating toilsome investigations, terminating in the same results, as what you now obtain more easily and expeditiously; and it gives you starting-places from which you may, if so inclined, push onwards in new careers of invention. It is true, that many powerful minds have allowed themselves to become entangled in the mazes of the mere literature of science, when they ought to have been labouring in fields of observation. You will do wisely to take warning from such examples; but, on the other hand, beware of falling into a habit of despising recorded facts and opinions; and of imagining, that all which is necessary to be learnt you can teach yourselves; and of listening to that cant which tells you to rely, solely, upon your own experience, and to allow of no guide but nature." 21.

The medical student, and the medical man must be ever a student, should exercise discrimination in the works he peruses. Dr. Symonds advises, and we perfectly concur with him, that those should be read first, which present a good summary of what is known on each particular subject—that then the history of discoveries and opinions in relation to it may be studied—and, finally, that the peculiar views of individuals may be carefully examined. Such a course of reading is the most complete that diligence could desire, or time and opportunity afford; but its very completeness precludes the idea of its being generally followed out. If the ordinary student makes himself conversant with the best summaries of what is known, he does as much as can reasonably be expected, and more, we are afraid, than is usually done.

Dr. Symonds reprobates the extreme of admiration or contempt for antiquity, an exaggerated love of novelty, or dread of innovation. He paraphrases the opinions of Lord Bacon—opinions still just, still applicable, still worthy of attention. In that monument of learning, dedicated to the advancement of it, Lord Bacon points out with a master's hand these conspicuous mental infirmities.

"The first of these peccant humours," says he, "is the extreme affecting of two extremities: the one antiquity, the other novelty; wherein it seemeth the children of time do take after the nature and malice of the father. For as he devoureth his children, so one of them seeketh to devour and suppress the other, while antiquity envieth there should be new additions, and novelty cannot be content to add, but it must deface; surely the advice of the prophet is the true direction in this matter, 'State super vias antiquas, et videte quænam sit via recta, et bona, et ambulate in ea.'"

"Another error, induced by the former, is a distrust that any thing should be now to be found out, which the world should have missed and passed over so long time; as if the same objection were to be made to time that Lucian maketh to Jupiter, and other the heathen gods, of which he wondereth that they begot so many children in old time, and begot none in his time; and asketh whether they were become septuagenary, or whether the law Papia, made against old men's marriages, had restrained them. So it seemeth men doubt, lest time is become past children and generation; wherein, contrariwise, we see commonly the levity and unconstancy of men's judgments, which, till a matter be done, wonder that it can be done; and, as soon as it is done, wonder again that it was no sooner done; as we see in the expedition of Alexander into Asia, which at first was prejudged as a vast and impossible enterprise: and yet afterwards it pleaseth Livy to make no more of it than this; 'Nil aliud, quam bene ausus est vana contemnere:' and the same happened to Columbus in the western navigation. But in intellectual matters, it is much more common; as may be seen in most of the propositions of Euclid, which, till they be demonstrated, they seem strange to our assent: but, being demonstrated, our mind accepteth of them by a kind of relation, as the lawyers speak, as if we had known them before." 41.

It must be admitted that the fault of our day is *not* a reverence for antiquity. So many great improvements have occurred, so much has been effected in the science and in the practice of medicine, that the stability of the professional mind has been disturbed, and, in its vertigo, every new object presented to its vision wears a distorted and exaggerated shape. We can scarcely open a journal, without finding some preposterous proposal, confidently recommended or magisterially enforced, and sure to hook a goodly number of simple-witted readers.

2. Lectures were formerly thought to be another method of conferring and of receiving instruction. Dr. Symonds is so far behind the level of the day, that he still continues to entertain a sentiment which men of liberality and spirit have discarded. His obsolete ideas are thus expressed.

"To some or all of you, it may possibly appear that, excepting those which are accompanied by demonstrations and experiments, lectures present no greater advantage than can be derived from books. But, before noticing this objection, I may remark, that they are necessary in the present state of things; that is, an attendance upon them is imposed by those who have the duty of ascertaining the qualifications of aspirants to practice. The reason for their imposition is, probably, not only a conviction of their use in medical education, but the want, at present, of any better proof that a certain quantity of time and attention have been devoted to study. But independently of their being required, they present many obvious advantages. The subjects may be treated more amply, more minutely, and more familiarly, than in books; and they may be illustrated by valuable experience, which would never have been communicated in any other form. Many persons also receive ideas, orally delivered, with less fatigue than by reading; and all must occasionally find it an agreeable change of occupation, to study by the ear instead of the eye. '*Levat lassitudinem etiam laboris mutatio.*' But we must not forget that the combination of the two modes of study affords advantages which cannot be derived from either, singly; one suggests further investigations; the other explains and throws new light upon the ideas first received." 23.

This may appear plausible, and on a superficial inquiry it may seem likely that physicians and surgeons who have devoted time and trouble to acquiring and to methodizing information, will possibly be enabled to convey some instruction to those young men who listen to their lectures. But such foggy notions are quickly chased away, when the new lights dawn on the medical horizon. We then discover that lectures are a juggle—prescribed courses of study a cheat—that young and inexperienced men are far the best judges of what they should or should not learn—far the best suited for guiding themselves—that parents, and masters, preceptors, and heads of colleges are all wrong, intolerant bigots, and rapacious oppressors, when they suppose or enjoin the necessity of any checks on the imprudence, improvidence, or want of mature wisdom of youth—in short, that young men should have their tether, and pick up information how they like and where they can.

It would be easy to enumerate the many salutary consequences which would spring from such a system. All the great schools would of course be broken up—lectures being unnecessary, and precariously attended, if at all, the many able men in London, Dublin, Edinburgh, and the great provincial towns, who now devote their time and talents to this method of instruction, would turn to other occupations—chemistry, anatomy, materia medica, and other sections of medical science, now absurdly looked on as demonstrative, would be studied much better without laboratories, musea, dissecting-rooms, injections, collections of drugs, or herbaria—each young man would exhibit the delightful spectacle of self-taught genius, and in short, we should return to those good old times when there were no establishments for medical education, when appreticeships flourished in septennial vigour, and when medical science was *so* much higher, than it is at present under the ridiculous and debasing system that prevails.

It has long been the opinion of those who are somewhat interested in the matter, nay of those who have surreptitiously passed for philosophers, that the passions of youth are too impetuous to allow it to dispense altogether with restraint. Experience has appeared to justify the sentiment, and in our own profession many have thought they discovered evidence of its correctness. Young men, who have left the houses of their fathers or their masters, for the purposes of study at universities or in towns, have often seemed to betray an ignorance of their real interests, and seduced by the contagion of example, or by the blandishments of passion, they have neglected the opportunities of acquiring knowledge,—absented themselves from lectures, dissecting-rooms, and hospitals—squandered their allowance at the theatre, the tavern, the billiard-room, and brothel—and returned to their relatives with minds and bodies debauched by dissipation, corrupted by vice, but not instructed in that professional knowledge they were dispatched in quest of. There are few of us who may not imagine that such cases are too common, who may not think that the constituted professional authorities are consulting the fondest wishes of parents, and the real interests of students, by exercising a judicious degree of vigilance, and by ensuring, so far as their regulations can, that attendance on prescribed lectures, which is professed, and which was thought necessary, or they would not have been prescribed at all.

But again we are set right by those who are imbued with the spirit of the age. Youth needs no kind of restraint—as it was capable of acquiring knowledge without the aid of lecturers or of schools, so it is fully equal to its own moral guidance—stringent examinations are tyranny—certificates of prescribed attendance are humbug—and any regulations to secure such attendance are a gross interference with the liberty of the pupil.

We begin ourselves to be infected with a noble enthusiasm for universal freedom of all sorts—we begin to see the villainous principles which have ever actuated fathers, and masters, and colleges, and governments—we begin to blush at the despotism exercised on youth, on that age which dotards conceive to want experience. But why stop at students? There is a much afflicted class, who may claim our sympathies for the wrongs that they endure—we mean the school-boys. Our blood boils when we think of the restraints put on *them*—of their being compelled to learn—of their little tender bottoms lacerated by " bummers." We can look on this harrowing picture no more. Down with all compulsory education—up with the voluntary system. Our creed is that of poor Grimaldi. When the mobs went about London, smashing the windows of all who did not chalk on their doors " No Popery," that most intellectual of clowns put up a board, on which was written " No Nothing." The good effects of the Saturnalia that are coming, are perhaps apparent in some degree already. After a broken head or two from the new police, a display at the Sessions, an indictment under Lord Ellenborough's Act at the Old Bailey, a personal inspection of the colony in New South Wales, all for the sake of maintaining the respectability of our learned profession, and, like Admiral Byng's execution, "pour encourager les autres," the modern neophyte will return to his admiring family a *finished* student.

3. Dr. Symonds concludes, and we quite coincide with him, that the *observation* of the student himself is more important than either lectures or reading. The object of each of the latter is to direct and to assist the former—to tell the young man what he is to look for, and how he may best find it. Lord Bacon, we believe, in his Essays remarks, that conversation makes a ready man, reading a learned man, and practice an expert man. In our own profession, observation alone makes a practical man. The spirit of observation and the habit of it should be warmly inculcated on the student, a spirit and a habit which will stick to him through life, and prove of incalculable service both to his mind and to his interests. Some of the men who have adorned medicine most, have displayed this taste for observation in a striking manner. It was either Petit or Paré of whom the following anecdote is told. An operation of some consequence was to be performed early in the ensuing morning at the Hôtel Dieu. When the attendants proceeded to open the operating theatre, they found young Paré on the staircase, where he had passed the night, in order to obtain a good view of the operation when the doors were unbarred. An omen of the future practical distinction of the man. For our own parts we would rather trust our lives to a man who had read little, heard little, but had observed long and carefully in a large hospital, than to the most accomplished scholar of the profession, who had had no opportunities of actual observation. Fortunately for the youth of our times, both the metropolis and the provincial towns present the combination of all modes of instruction—good libraries, good lectures, and well-appointed hospitals. When his elementary knowledge is obtained, the student should repair to the wards of the hospital, and watch and note the cases it contains. There is a rich array of facts, there he acquires that relish for them, so characteristic of a manly mental appetite, there he forms the habit of thinking and of acting, which in after-life will give him confidence when less observant men will be confounded, and will confer on him those qualities so requisite for professional success—decision, accuracy, and the command of resources.

### 3. *The Spirit of Professional Study.*

What signifies the mode if the spirit of study is not good? That gives a tone to all that is done, and, like *conduct* in life, may make or mar success, in spite of talent, or without it.

Dr. Symonds inculcates the necessity of a passion for medicine, at all events of a taste for it—of the inclination and the habit of making its objects our first object—of patience in the pursuits of its truths and of their reward. We may say to the hesitating and discouraged student what was said to that young lawyer who afterwards held with such high reputation the great seal of England, "Go on, John, turn neither to the right nor left, and you'll be Lord Chancellor at last." To proceed with Dr. Symonds' injunctions, he does not forget to enumerate diffidence in forming an opinion, a diffidence so characteristic of knowledge, so frequently contemned by ignorance—nor does he omit insisting on what is sometimes disregarded, the entertainment of a deep respect for the profession of which we are members. This is a very excellent precept. How can we expect men to attach credit to either our science or ourselves, when we affect a scepticism of its powers? Who would listen to the exhortations of that Christian minister, who openly avowed himself a Deist, and derided the crucifix that he called on men to reverence? Who could feel confidence in the treatment of the physician, who doubted the utility of those measures he employed—measures which, in accordance with his own principles, should be inert, or, if active, must be hazardous. It may be confidently said, that the physician who expresses doubts of the powers of medicine displays the extreme either of ignorance or folly—of ignorance if he is sincere—of folly if he is not. The application and the efficiency of medicine in a special malady may admit of doubt; its utility, as a broad proposition, can, to rational and practical men, admit of none.

And what are the prizes which will tempt the sickly student to inhale the foul atmosphere of the ward and of the dead-house, to attend unremittingly the lecture and dissecting room, and to deprive himself of that sweet sleep which rests on the mechanic's pillow, in order that he may pore on the scientific page? Worldly distinction is not one of those prizes. The fame which the Gazette confers is not for him—he has not, like the advocate, the prospect of the ermine, the seals, or the coronet—medicine has not, with the Church, her lawn sleeves—nor can she, with successful trade, pour thousands in the lap. What then *does* she offer? It must be owned that to estimate her favours highly, it is necessary to weigh them in a philosophic balance. Industry may reasonably look forward to a competence, and may anticipate crowning its "life of labour by an age of ease." But prudence will not count on more, and science will whisper, "look to me for your reward." That reward consists in the love of philosophic truth, and in the consciousness of ever pursuing and at times attaining it—in the conviction that our profession is stamped by reason, by custom, and by God, as a benevolent and noble one in principle and practice—in the reflection that its exercise improves our mental and develops our moral faculties, brings us into relation with all classes, and that sort of relation which links us with their fate, and endears us to their sympathies,—and, finally, in the experience which it enables us to acquire of the sober pleasures of domestic life. Ambition spurns the tame picture we have drawn, and leaves to philosophy a profession which has only the mean and contemptible privilege of rendering a wise and a good man happy.

